# An AAV-Based Therapy Approach for Neurological Phenotypes of X-Linked Adrenoleukodystrophy

**DOI:** 10.3390/ijms262311645

**Published:** 2025-12-01

**Authors:** Ekaterina Gornostal, Almaqdad Alsalloum, Egor Degtyarev, Ekaterina Kuznetsova, Aygun Levashova, Daria Mishina, Natalia Mingaleva, Ali Mazloum, Viktor Bogdanov, Julia Krupinova, Sergey Mikhalkov, Irina Rybkina, Olga Mityaeva, Pavel Volchkov

**Affiliations:** 1 Federal State Budgetary Scientific Institution “Federal Research Center for Innovator and Emerging Biomedical and Pharmaceutical Technologies”, 125315 Moscow, Russia; gornostal.kate@gmail.com (E.G.); alsallum_a@academpharm.ru (A.A.); mazlum.a@genlab.llc (A.M.);; 2 Moscow Center for Advanced Studies, Kulakova Str. 20, 123592 Moscow, Russia; 3 Loginov Moscow Clinical Scientific Center, 111123 Moscow, Russia; 4 Department of Endocrinology, Morozov Children’s Clinical Hospital, 119049 Moscow, Russia; 5 Department of Fundamental Medicine, Lomonosov Moscow State University, 119992 Moscow, Russia

**Keywords:** X-linked adrenoleukodystrophy, X-ALD, demyelinating disorders, AAV, gene therapy

## Abstract

X-linked adrenoleukodystrophy (X-ALD) is a monogenic progressive neurodegenerative disorder, being simultaneously a systemic metabolic disease and demonstrating severe neurological manifestations with effects to the brain and spinal cord. The objective of the current review is to provide a detailed approach to adeno-associated virus (AAV)-based gene therapy for neurological manifestations of X-ALD. The development of a successful AAV-mediated gene therapy hinges on its ability to deliver *ABCD1* cDNA effectively to the relevant organs and cell types, induce therapeutic levels of protein expression, and ultimately, restore normal very-long chain fatty acids (VLCFA) metabolic function. Thus, several key considerations should be addressed when designing AAV-based gene therapy for X-ALD, including the genetic background of the disease and requisite transgene expression levels, the biochemical function of the adrenoleukodystrophy protein (ALDP), the identification of target cells and their role in pathogenesis, the regulation of expression within the genetic construct, the route of administration, the selection of an AAV serotype with high tropism for the central and peripheral nervous systems, and the development of robust in vitro and in vivo models.

## 1. Introduction

X-linked adrenoleukodystrophy (X-ALD) is a progressive metabolic disorder characterized by the involvement of the adrenal cortex and the nervous system, including both central and peripheral components. While its estimated incidence is approximately 1 in 17,000 in males and heterozygous females, the true prevalence is likely to be higher [1,2]. The disease is associated with pathogenic variants in the *ABCD1* gene, which encodes the adrenoleukodystrophy protein (ALDP), also known as ATP-binding cassette sub-family D member 1. ALDP facilitates the peroxisomal import of saturated and monounsaturated very long-chain fatty acids (VLCFA). Dysfunction of this protein results in impaired peroxisomal β-oxidation and the accumulation of VLCFAs in cytosol.

The manifestations of X-ALD present with significant variability, and three main phenotypes are distinguished.

Adrenomyeloneuropathy (AMN) is characterized by dying-back axonopathy, leading to a range of neurological manifestations. These include chronic pain, spastic paraparesis, bladder and bowel incontinence, and sexual dysfunction. The disease manifests with a pronounced sex-specific disparity in onset and prevalence. In hemizygous males, symptom onset typically occurs between the second and third decades of life (ages 20–30), with disease progression plateauing after approximately 15 years. AMN represents the most common adult manifestation of X-ALD in males, with a relative frequency of 40–46%. In contrast, heterozygous females present later, with onset usually between the fourth and fifth decades (ages 40–50), and for them, AMN is the primary and often only clinical manifestation of X-ALD [3].

Cerebral X-ALD (cALD) is a rapidly progressive inflammatory demyelination, most often occurring in the corpus callosum and steadily progressing to a symmetrical, confluent lesion in both cerebral hemispheres. cALD manifests predominantly in childhood, with onset never occurring before 2.5 years of age, and rarely in adolescents and adults [4]. The progression of the disease is characterized by neurological deterioration, which can ultimately result in a vegetative state or death within a period of 3–5 years.

It has been determined that 70% of males diagnosed with X-ALD are also affected by primary adrenal insufficiency, also known as Addison’s disease. The condition is most frequently linked to cerebral ALD or AMN phenotypes. Nevertheless, a certain number of patients suffering from adrenal insufficiency do not exhibit overt neurological abnormalities. In such cases, the diagnosis is labelled as “Addison’s disease only” in X-ALD, but such patients may also develop neurological manifestations over time. The clinical manifestations of isolated adrenal insufficiency in X-ALD are no different from those of Addison’s disease caused by other etiologies, but the diagnosis can be confirmed by elevated plasma VLCFA levels [5].

One of the salient features of X-ALD is the absence of correlation between phenotype and genotype [4]. This is evidenced by the observation that even members of the same family are more likely to exhibit divergent manifestations of the disease; moreover, different phenotypes can overlap over the course of the disease [6].

Therapeutic approaches for the treatment of X-linked adrenoleukodystrophy remain challenging due to the disease’s phenotypic complexity and the absence of therapies capable of achieving long-term genetic correction. Current interventions are phenotype-specific. While primary adrenal insufficiency is effectively managed with hormone replacement therapy, no disease-modifying treatments exist for AMN, for which care remains solely supportive. In the cerebral form, the process of inflammatory demyelination can be halted by means of hematopoietic stem cell transplantation (HSCT) [7]. The efficacy of this intervention is based on a unique biological mechanism: following transplantation, donor-derived cells repopulate the brain’s resident immune cells (microglia). These healthy, donor-derived microglia express a functional ABCD1 protein, which enables them to metabolize VLCFAs normally. Through a process of metabolic cross-correction, they restore normal lipid metabolism in the central nervous system environment, thereby quenching the neuroinflammatory cascade and stabilizing cerebral demyelinating lesions [8]. The success of this intervention is highly time-sensitive, as it is contingent upon early diagnosis and intervention prior to the establishment of irreversible neurological damage. However, timely diagnosis is often complicated by the initially subtle or nonspecific nature of symptoms, which only become more pronounced after significant demyelination lesions have occurred [9]. Newborn screening could provide early detection, which would allow for prompt follow-up and treatment to prevent serious complications [10]. Despite the fact that HSCT improves the course of the disease in most cases, it is associated with serious risks, such as graft-versus-host disease, graft failure, infection, or rejection [11].

In response to the rising need for the creation of new more effective and safe treatment, several approaches have been studied. Gene therapy has been identified as a potential solution for X-ALD. The rationale for this approach was established as early as 1994, when researchers determined that the disease fulfills the criteria for a successful gene therapy candidate [12]. In vitro studies demonstrated that a recombinant retrovirus successfully infected skin fibroblasts from childhood cALD and AMN patients with the *ABCD1* gene, subsequently resulting in the normalisation of VLCFA levels in the treated cells [13].

The foundational gene therapy strategy for X-ALD comprises the harvest of autologous hematopoietic stem cells (HSCs), their ex vivo genetic modification, and subsequent reinfusion into the patient. The implementation of this approach is prominent in the domain of gene therapy, specifically in the form of Lenti-D gene therapy (elivaldgene autotemcel/eli-cel). The basis of this treatment is the intravenous administration of patient’s hematopoietic stem cells (HSC) that have been transduced with a lentiviral vector containing a functional copy of the *ABCD1* gene. The modified HSCs are transplanted into patients only after prior myeloablative preparation (NCT01896102). This therapeutic approach is particularly beneficial in the early stages of cALD, especially for patients lacking a suitable matched donor. In cases of rapidly progressive cerebral ALD, delays in securing a donor can critically impact the patient’s prognosis. However, it is important to note that this therapy is not effective for patients with spinal cord involvement (AMN). While Lenti-D can effectively stabilize neurological decline when administered early, this benefit is counterbalanced by significant safety risks. The most critical of these is insertional mutagenesis, where the semi-random integration of the lentiviral vector can disrupt tumor suppressor genes or activate proto-oncogenes, potentially leading to clonal expansion and malignancy [14,15,16,17,18]. This theoretical risk has been confirmed in clinical follow-up, with recent long-term data indicating that approximately 10% of treated patients developed hematologic malignancies, including cases of myelodysplastic syndrome and acute myeloid leukemia [19,20,21].

The second approach is in vivo gene therapy, i.e., the delivery of a functional copy of a gene directly into the patient’s system. Clinical trials are currently investigating therapeutic agents based on this strategy. The pharmaceutical compound Lentiviral IT-IV is a self-inactivating lentiviral vector that carries a functional copy of *ABCD1*, which is administered either via intrathecal or intravenous injection (NCT03727555). Lentiviral vectors undoubtedly have certain advantages, including a large packaging capacity (8–12 kb), the ability to infect both dividing and non-dividing cells and relatively low cost of production when compared to alternative systems, such as adeno-associated virus (AAV) [22]. To date, however, there are no FDA-approved in vivo gene therapies based on lentiviral delivery.

The third pharmaceutical compound under investigation is the AAV-based SBT101, which was being evaluated in clinical trials (Spur Therapeutics, Inc, New York, NY, USA). Although this specific trial was terminated (as of 29 October 2025; NCT05394064), the therapeutic approach involved the administration of a recombinant adeno-associated virus serotype 9 (AAV9), which contains a functional copy of the *hABCD1* under a CAG promoter, to adults with AMN through an intrathecal route. The advantage of choosing such a vector is that AAV does not integrate into the host genome, but delivers the coding sequence of a gene into the nucleus, where the transgene exists as an episome, ensuring long-term expression in non-dividing cells. For targeted transgene delivery, it is possible not only to select the most suitable serotype, but also to design novel capsid variants with desired tropism and lower immunogenicity [23]. These characteristics continue to render AAV-mediated delivery a promising therapeutic strategy for the treatment of genetic disorders such as X-ALD.

In the case of X-ALD, it is challenging to develop a universal therapy that would be effective for all phenotypes. The current inability to effectively and specifically target both the adrenal glands and the nervous system suggests that a more viable strategy may be to develop distinct therapeutic interventions for the adrenal and neurological manifestations separately.

**Figure 1:**
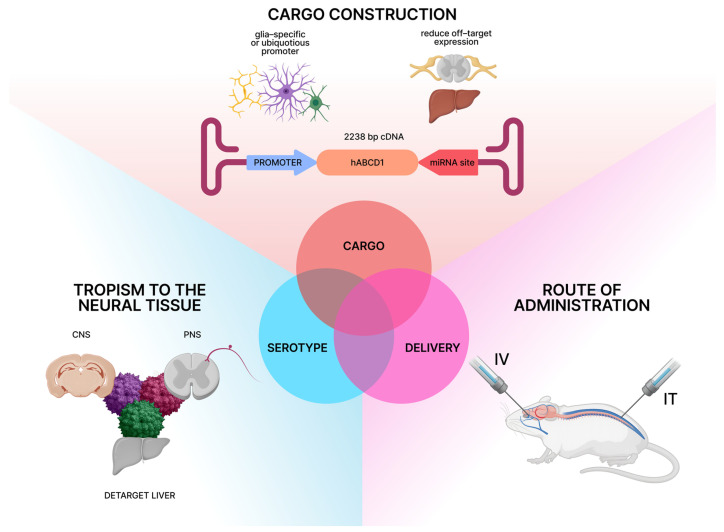
Crucial components of AAV-based gene therapy for neurological manifestations of X-linked adrenoleukodystrophy (Created in Figma Desktop App version 125.9.10. Ekaterina Gornostal. (2025)).

This review focuses on an approach to AAV-based gene therapy for neurological phenotypes of X-linked adrenoleukodystrophy, informed by recent advances in understanding its pathophysiology. The development of an effective AAV-based gene therapy for the neurological phenotypes of X-linked adrenoleukodystrophy (X-ALD) relies on three fundamental components. First, the AAV capsid, which determines tropism in central and peripheral nervous systems with lower affinity to the liver to avoid side effects associated with toxicity. Second, the genetic cargo, consisting of a functional copy of *hABCD1* with key regulatory elements. These include either ubiquitous or cell-specific promoters and enhancers, as well as miRNA target sites for deliverance of off-target expression. Third, the delivery method, such as intrathecal (IT) or intravenous (IV), which determines the region-specific transgene expression, being equally effective and safe (Figure 1). The primary design of the therapy must also rely on the model systems (in vitro and in vivo) applied in preclinical studies to correctly assess the therapeutic effect.

## 2. The ABCD1 Gene, Lipidomic Dysregulation, and Pathological Mechanisms in X-ALD

### 2.1. ABCD1: Gene, Coding Sequence, Protein Structure and Function

The *ABCD1* (NCBI NC_000023.11) gene is located on the long arm of the X chromosome at cytogenetic band Xq28. In the GRCh38.p14 human genome assembly, *ABCD1* encompasses approximately 19.9 kilobases of genomic DNA and is organized into 10 exons. The gene produces a transcript represented by the mRNA (NCBI NM_000033.4) of 3669 nucleotides in length, containing all 10 exons, with a coding sequence (CDS) (NCBI CCDS14728.1) spanning 2238 nucleotides. This gene encodes the ATP-binding cassette subfamily D member 1 protein (ABCD1), also known as adrenoleukodystrophy protein (ALDP), which consists of 745 amino acid residues (UniProt P33897). ABCD1 belongs to the large family of membrane proteins that utilize ATP hydrolysis energy to transport various substrates across cellular membranes which are called ABC transporters [24]. Within its subfamily, ABCD1 shows 65% and 40% sequence identity with ABCD2 and ABCD3, respectively [25]. These three human peroxisomal ABC transporters play a vital role in importing lipid substrates into peroxisomes for degradation via β-oxidation [26]. The protein functions as a half-transporter containing two main domains: a transmembrane domain (TMD) with six hydrophobic α-helical segments spanning the peroxisomal membrane, and a nucleotide-binding domain (NBD). ABCD1 requires homodimerization to form a functional transport unit, though heterodimers with ABCD2 and ABCD3 have been reported, with functional homodimers appearing most prevalent in vivo [27,28,29]. It remains unclear whether the oligomerization of peroxisomal ABC transporters has any influence on substrate specificity [30]. The protein is widely expressed and has high substrate specificity. β-oxidation represents a conserved peroxisomal process by which acyl groups are degraded two carbons at a time, with very long-chain fatty acids (VLCFAs, >22 carbons) being exclusively β-oxidized in peroxisomes, making this organelle essential, especially in brain tissues [31].

Pathogenic variants in the *ABCD1* gene cause a peroxisomal disorder called X-linked adrenoleukodystrophy (X-ALD), with an average incidence in males of 1:20,000 [3]. At present, 1215 unique variants/cases are described in the X-ALD database (https://adrenoleukodystrophy.info/mutations-biochemistry/mutation-statistics, accessed on 22 September 2025). The predominant types of gene variant are missense (53.31%), nonsense (10.47%), frameshift (23.58%), amino acid insertion/deletion (4.55%), and one or more exons deleted (1.99%). Deletion, frameshift, and nonsense mutations produce truncated ABCD1 proteins. In contrast, missense variants often lead to protein instability and reduced abundance. It seems likely that an ABCD1 protein with a missense mutation undergoes incorrect folding, mis-targeting, and an impaired ability to dimerize. These defects collectively contribute to the degradation of the mutant protein. Pathogenic variants in *ABCD1* are predominantly located in the transmembrane domain (46%, exons 1–2) and ATP-binding domain (35%, exons 6–9). The highest density of pathogenic variants is observed in exon 1, followed by exon 6, with comparable densities in exons 2, 8, and 9. Exon 1 contains the highest number of pathogenic variants, which are distributed over the last two-thirds of the exon (amino acids 100–300).

X-ALD shows a wide range of phenotypic expressions. Different clinical manifestations often co-occur within a single family, with phenotypic heterogeneity being more common than uniformity among affected kindreds [3,32]. Furthermore, no consistent genotype–phenotype correlation has been established, as the clinical outcome does not reliably predict either the specific *ABCD1* pathogenic variant or the level of ALDP expression.

### 2.2. Lipidomics Associated with Defective ABCD1

The major biochemical feature of X-ALD is an accumulation of very long-chain fatty acids (VLCFA) due to the dysfunction of ABCD1 protein. When proper translocation by transporter is impaired, VLCFAs are enriched in the cytosol in various esters. VLCFAs accumulate in all tissues and organs, but severe pathological consequences appear only in the brain, spinal cord and peripheral nerves, adrenal glands, and testis [33,34,35,36]. The role of VLCFA accumulation in pathogenesis still remains unclear. Unlike long-chain fatty acids, VLCFAs differ in physiological characteristics mostly due to their high hydrophobicity. This may drastically affect metabolic processes and membrane stability in which they are incorporated. VLCFAs are not inherently toxic, but their incorporation into complex lipids, such as cholesterol esters (CE), phosphatidylcholine (PC), or triglycerides (TG) disrupts membranes, generates toxic metabolites, and triggers oxidative stress, endoplasmic reticulum (ER) stress, and inflammation [37,38,39,40,41,42,43]. The concentration of free form VLCFAs in plasma (C26:0 levels, C24:0/C22:0 and C26:0/C22:0) is one of the golden standard biomarker in X-ALD diagnostics, despite the fact that it does not reflect disease progression and development of a certain phenotype. The early research failed to find any correlation between disease severity and VLCFA levels [5,44]. These studies focused only on the free form concentration of VLCFAs in plasma of patients, losing information about the different lipid classes they can be incorporated in. The early clue about lipidomic peculiarities that could be associated with the course of the disease appeared when post mortem brain tissues of patients with cerebral phenotype were analyzed. In active demyelination lesions VLCFAs strongly dominate in CE, which are believed to be accumulated in monocytes and macrophages after disruption of the blood–brain barrier (BBB) [45]. In both normal-appearing white matter and demyelinating areas VLCFAs are enriched in PC fraction [46,47]. Thus, accumulation of enriched PC might forerun demyelination. It is shown that lysophosphatidylcholine (LPC) containing VLCFAs are cytotoxic and induce microglial apoptosis [48].

Although VLCFA-containing lipids have been well-documented in ALD, their broader impact on the global lipidome—and how these alterations influence disease severity and progression—remains poorly understood. Lipidomics presents a powerful tool to address these gaps, enabling a systems-level exploration of the metabolic disturbances underlying ALD pathogenesis.

In a large cohort study, Jaspers et al. utilised a lipidomic approach to find a strong correlation between lipidome of patients with X-ALD and course of the disease [49]. Among all VLCFA-enriched lipid classes LPC, TG, and PC fractions were the most elevated in ALD patients in comparison with healthy donors. Notably, LPC C32:0 and PC C46:4 levels were the highest in patients with cerebral ALD when compared with patients with no cerebral involvement. Further research is needed to estimate these lipid fractions as novel potential biomarkers. In patients with severe spinal cord disease VLCFA-enriched lipids were estimated higher than in samples obtained from the cohort with mild spinal cord disease. Elevated levels in severe AMN phenotype could indicate ongoing lipid toxicity driving neurodegeneration.

Although research has advanced our understanding of ALD, the exact mechanisms by which VLCFA accumulation drives disease progression and influences its diverse clinical manifestations remain unclear.

### 2.3. Lipidomics Draws a Link Between the Pathological Processes of Neuroinflammation and Neurodegeneration in X-ALD

Despite the fact that pathogenic variants in the *ABCD1* gene cause systemic biochemical defect, the most severe pathological consequences affect predominantly glial cells in CNS and PNS, adrenal glands (the zona fasciculata and zona reticularis in the cortex) and Leydig cells in testis.

In terms of neurological phenotypes, the accumulation of VLCFA-enriched lipid fractions—particularly lysophosphatidylcholine (LPC C32:0, LPC C26:0), phosphatidylcholine (PC 46:4), and triglycerides (TG)—in X-ALD may contribute to disease pathogenesis through several mechanisms. LPC is a pro-inflammatory lipid that can disrupt membrane stability and induce cytotoxicity [50,51]. Elevated LPC may activate microglia and astrocytes, promoting neuroinflammation—a key feature of cerebral ALD [52,53]. LPC is a known damage-associated molecular pattern (DAMP) that can trigger inflammasome activation, notably of the NLRP3 complex. It was demonstrated that NLRP3 is active in demyelinating lesions in post-mortem tissues of cALD patients [54,55]. Furthermore, the elevated lipid species PC 46:4, if structurally altered by the incorporation of VLCFA, may be recognized as “non-self” antigen, potentially further driving autoimmune-like responses [56].

It was shown that VLCFA-enriched lipids in the spinal cord can lead to oxidative stress, increased reactive oxygen species (ROS) production, damaging neurons via impaired ATP-dependent axonal transport [39]. Moreover, accumulation of lipids, detected on ultrastructural level in dorsal root ganglion of AMN patients, in neuronal mitochondria can contribute to the dying-back axonopathy [57].

In both human and mouse AMN spinal cords, phagocytosis-related markers (e.g., MFGE8, TREM2) are upregulated before complement activation and synapse loss, notably in the absence of significant inflammation [58]. This suggests a distinct, inflammation-independent pathway of neurodegeneration. Supporting this mechanism, ABCD1-deficient microglia exposed to LPC C26:0 upregulate MFGE8, amplifying their phagocytic activity and resulting in neuronal injury. Thus, toxic lipid accumulation—a hallmark of AMN—directly triggers aberrant microglial phagocytosis, which contributes to disease progression.

The VLCFA-enriched LPC and PC species are likely contributors to X-ALD pathogenesis, promoting neuroinflammation, membrane dysfunction, oxidative stress, and mitochondrial failure. Their strong correlation with disease severity implies a pathogenic role, indicating that these lipids are not merely passive biomarkers but central drivers of neurodegeneration in both cerebral ALD and spinal cord disease. Further studies are needed to explore the therapeutic potential of modulating these lipid fractions by delivering a functional copy of *hABCD1* to affected cells. Furthermore, we propose that monitoring the lipidomic profile—specifically LPC, PC and TG species—rather than total VLCFA concentration, provides a more physiologically relevant biomarker for assessing treatment response. In this matter, additional research on mouse models, such as *Abcd1^−^/Y* knock-out and *Abcd1^−^/Y*; *Abcd2^−^/^−^* double knock-out mice, is necessary to evaluate efficacy of anticipation of preclinical trials.

## 3. Determinants of the *ABCD1* Expression Cassette for AAV-Based Gene Therapy

Gene therapy relies on precise control of gene expression, whether to modulate mRNA levels or target specific tissues. This section explores current strategies for regulating gene expression in therapeutic applications, focusing on two key approaches. The first strategy is transcriptional regulation using tissue-specific or ubiquitous promoters to direct spatial and temporal expression patterns. Another level is the post-transcriptional control through RNA interference (RNAi) techniques to regulate mRNA stability and translation in specific organs and tissues [59].

### 3.1. Defining Gene Therapy Targets in X-ALD

The application of modern single-cell and single-nucleus RNA sequencing (snRNA seq) has provided transformative insights into the expression patterns of the *ABCD1* gene. Although earlier studies successfully identified astrocytes and microglia as the primary *ABCD1*-expressing cells, particularly within white matter regions, their resolution was limited [60]. In contrast, contemporary single-cell technologies offer unprecedented understanding into cell-specific expression levels, regional differences, and co-expression with other genes. This advancement enables more precise therapeutic targeting and deeper understanding of cellular vulnerability in X-ALD.

**Figure 2:**
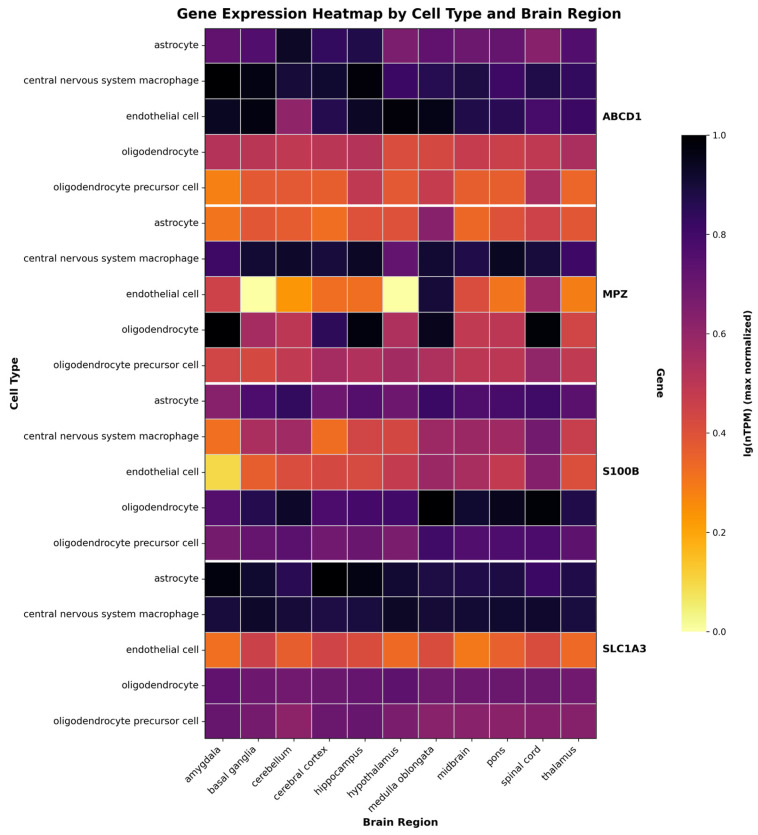
Gene expression patterns across brain regions and cell types. Heatmap showing expression of four genes (*ABCD1*, *MPZ*, *S100B*, *SLC1A3*) in six CNS cell types across eleven brain regions. Color intensity indicates expression level in nTPM max normalized across one cell type in one gene. Data derived from Human Brain Atlas (https://www.proteinatlas.org/humanproteome/single+cell/single+nuclei+brain/data#brain_regions, accessed on 22 September 2025) and visualized using custom code available at GitHub repository (https://github.com/EgorDegtyarevAP/gene-expression-heatmap.git, v1.0.0 (accessed on 22 September 2025)).

The selection of target cell types for X-ALD gene therapy requires a comprehensive understanding of both the natural expression pattern of *ABCD1* and the cellular mechanisms underlying disease pathogenesis. Recent transcriptome analyses from the Human Brain Cell Atlas have provided novel resolution of gene expression across different brain cell subtypes [61]. snRNA-seq data reveal that *ABCD1* is predominantly expressed in glial cell populations, with the highest expression levels observed in microglia, followed by astrocytes, endothelial cells, oligodendrocytes and oligodendrocyte precursor cells (OPC) (Figure 2).

Microglia constitute the central therapeutic priority for X-ALD due to its high *ABCD1* expression and pivotal role in neuronal homeostasis and inflammatory regulation, making them particularly vulnerable to the toxic effects of accumulating VLCFA as they actively express *ABCD1* in human and mouse brain [60]. These cells maintain brain function through multiple critical processes including immune surveillance, synaptic pruning, and inflammatory regulation, relying on proper lipid metabolism to support their high phagocytic activity [62]. Recent histopathological analyses reveal that microglia damage precedes major myelin breakdown, with dramatic microglia loss occurring in prelesional areas where remaining cells completely lose homeostatic markers and adopt an amoeboid morphology, while oligodendrocytes and myelin remain largely intact. When microglia are incapable of metabolizing accumulated VLCFA, they undergo a unique form of programmed cell death. This process is characterized by diffuse cytoplasmic DNA fragmentation in the absence of classical apoptotic or pyroptotic markers, occurs in an immunologically silent manner, and results in the loss of their essential protective functions [63]. Since neurons depend on microglial support for synaptic maintenance and debris clearance, the loss of functional microglia initiates a cascade of neuronal vulnerability. Experimental evidence demonstrates that VLCFAs exert direct cytotoxic effects on microglia, inducing cell death at the periphery of actively demyelinating lesions. The subsequent rupture of dying microglia releases their contents, including undegraded, VLCFA-enriched myelin fragments, thereby triggering inflammatory cascades and recruiting peripheral immune cells [48]. Restoring ABCD1 function in microglia is therefore essential to prevent neuroinflammation and preserve immune homeostasis within the balance.

Astrocytes are another primary target due to their high *ABCD1* expression and crucial roles in BBB function and neuronal support. These cells maintain neuronal function through multiple processes including nutrient supply, lipid metabolism, neurotransmitter regulation, and antioxidant protection [64]. To meet their substantial energy requirements, these cells rely primarily on fatty acid oxidation, which generates acetyl-CoA for metabolic processes [65]. Astrocytes additionally capture surplus fatty acids released by hyperactive neurons via ApoE-containing particles, and store them intracellularly as lipid droplets [66]. When astrocytes cannot properly break down fatty acids, nearby neurons show impaired metabolism, reduced synapses, and increased vulnerability to inflammation and degeneration [67]. Due to their limited capacity for fatty acids catabolism, neurons are vulnerable to lipotoxicity, wherein lipid accumulation promotes the generation of harmful reactive oxygen species and induces membrane damage [66,68]. Furthermore, astrocyte-mediated support is also compromised by lipid related deficits, which harms synapse development and myelination [69,70]. This link is starkly evident in the astrocytes that undergo a transition to a reactive state and release inflammatory molecules [71]. Complementary in vitro studies utilizing patient-derived pluripotent stem cells (iPSC) models confirm that ALD astrocytes exhibit a profoundly dysregulated lipidomic profile and impaired functional capacity to provide neuronal support. These findings collectively demonstrate that loss of ABCD1 function has cell-autonomous consequences, directly compromising core astrocyte functions [72].

Oligodendrocytes represent essential cellular targets in the pathogenesis of ALD due to their dual role in both myelin production and their particular susceptibility to the toxicity of VLCFA. These cells express moderate *ABCD1* levels and generate the lipid-rich myelin sheaths that insulate axons within the CNS. The high metabolic demands of myelin production make oligodendrocytes particularly susceptible to toxic and inflammatory factors [73]. The inflammatory processes are predominantly directed against oligodendrocytes, ultimately leading to the progressive and widespread demyelination that defines the disease’s pathology [46,74]. In cALD a significant loss of oligodendrocytes was observed in the actively demyelinating zone, while in the gliotic zone they were entirely absent and displayed altered morphology with few or no processes in the gliotic zone. In the AMN spinal cord, the number of oligodendrocytes was also reduced [60]. Restoring ABCD1 function in oligodendrocytes is crucial for preventing myelin breakdown and supporting repair processes.

Endothelial cells are increasingly implicated in the pathogenesis of X-ALD. As the fundamental complement of the BBB and a site of high *ABCD1* expression, these cells are central to maintaining cerebral homeostasis. Histopathological evidence from cerebral ALD patients shows significant BBB damage, which facilitates the transmigration of inflammatory cells into the CNS [75]. These pathological changes are corroborated by in vitro experiments which demonstrate that ABCD1 loss reduces tight junction proteins that maintain barrier integrity [75]. Additionally, neuroimaging studies reveal that blood vessel disturbances occur before visible MRI signs of CNS demyelination [76]. In disease progression, areas with reduced blood flow develop inflammation, suggesting that vascular dysfunction precedes tissue damage. Importantly, stem cell transplantation can restore normal blood vessel function and barrier integrity, supporting the therapeutic importance of targeting endothelial cells [77].

The pathophysiology of X-ALD is driven by a dynamic crosstalk between four CNS cell types. Pathological changes within one cell compartment instigate a cascade of dysfunction in others, establishing a self-perpetuating cycle of damage. This vicious cycle is characterized by activated microglia triggering neuroinflammatory astrocytic response, while simultaneously, dying oligodendrocytes release cytotoxic myelin debris that further potentiates microglia activation [53,54,55]. Critically, endothelial dysfunction worsens this process by compromising BBB integrity, thereby facilitating the transmigration of peripheral immune cells and intensifying the inflammatory cascade and subsequent demyelination.

Building upon the preceding analysis, the rationale for targeting microglia, astrocytes, oligodendrocytes, and endothelial cells in X-ALD gene therapy is justified by their high *ABCD1* expression levels, as demonstrated by modern sequencing technologies, their central roles in disease progression through interconnected mechanisms, and their accessibility through cell-specific therapeutic approaches.

### 3.2. Determining Promoters for X-ALD Therapy Relying on a Gene Expression Patterns

Promoters are key elements that determine where and how actively a gene will be expressed. In the context of control under *ABCD1* expression, both ubiquitous and cell-type-specific promoters optimized for operation within the limited capacity of AAV vectors (~4.7 kb) are used [78]. Ubiquitous promoters can drive strong expression and are suitable for global compensation of protein deficiency in the case of systemic disorders, but they can cause overexpression of transgene in off-target cells, leading to side effects [79,80]. Tissue-specific promoters possess less transcriptional activity as they are used to fine-tune target expression, minimizing the side effects of overexpression and increasing the effectiveness of therapy in critically important cells [81,82,83].

Ubiquitous promoters are often derived from virus expression systems or composed of regulatory elements from highly expressed human genes. They ensure the expression of the transgene in the widest possible range of cells with evidence of transduction of glial cells [84]. Among the most frequently used constitutive promoters are cytomegalovirus (CMV), CMV enhancer the chicken beta actin promoter (CAG), simian virus 40 early (SV40), human ubiquitin C (UBC), human elongation factor 1α (EF1α), and human phosphoglycerate kinase 1 (PGK) [85,86].

In the context of X-ALD gene therapy, the use of constitutive promoters has several advantages, as their ubiquitous expression, compactness and strength make them ideal tools for correcting this systemic metabolic disease. The expression profile of *ABCD1* in the central nervous system underscores the need for widespread restoration of protein function [87,88]. Short and strong constitutive promoters offer the best balance between the packaging limitations of AAV vectors and therapeutic efficacy, allowing the use of lower doses of the vector to achieve the desired therapeutic effect [89,90,91]. A significant adverse effect of gene therapy employing constitutive promoters is their propensity to induce toxicity across various tissues. For instance, nonhuman primate (NHP) studies have documented histopathological evidence of neuroinflammation following treatment with AAV9 vectors driving GFP expression under the CMV promoter. This toxicity has been attributed to adaptive immune responses mounted against the ectopically expressed transgene product [92]. Separately, analysis revealed lesions within the dorsal root ganglia (DRG) of NHPs and mice. These lesions were characterized by neuronal degeneration and necrosis, increased cellularity, and nerve fiber degeneration, concomitant with elevated serum levels of phosphorylated neurofilament light chain (NFl), a biomarker of axonal injury [93,94,95].

In severe instances, toxicity in targeted tissues can precipitate the onset of ataxia [96,97,98]. The use of cell-specific promoters limits the expression of the transgene of interest in certain cells. Among the commonly used promoters for targeting glial cells in CNS: glial fibrillary acidic protein (GFAP) promoter provides astrocyte-specific expression, myelin basic protein (MBP) or the human myelin associated glycoprotein (MAG) promoter are specific to oligodendrocytes, and F4/80 promoter is used for targeting microglial cells [78]. These promoters are highly specific for their cell types and may not be suitable for multiple targeting in X-ALD therapy.

Based on the native expression profile of the *ABCD1* gene, we analyzed snRNA-seq data (https://www.proteinatlas.org/ENSG00000101986-ABCD1/single+cell/, accessed on 22 September 2025) [61] to identify genes with co-expression in the target cell populations. This analysis nominated the promoters of the *S100 beta*, *MPZ* and *SLC1A3* genes as potential candidates for constructing the expression cassette of the genetic cargo to target glial cells. Herein, we compile fundamental characteristics of these promoters, including their sizes and key regulatory elements that potentiate transcriptional activity.

The myelin protein zero (MPZ) promoter exhibits high selectivity for Schwann cells, the myelinating glia of the peripheral nervous system [99]. The MPZ protein is essential for myelin compaction, utilizing its adhesive properties to promote tight sheath-axon adhesion. Furthermore, it participates in signal transduction pathways critical for regulating myelin formation and maintenance, thereby ensuring the structural integrity and functionality of the myelin sheath [100,101]. MPZ minimal promoter is approximately 1.1 kb in length. Transcriptional regulation of the *MPZ* gene involves a critical enhancer element located within its first intron. This expression is directly modulated by key transcription factors, including SRY-box transcription factor 10 (SOX10) and early growth response 2 (EGR2) [102]. SOX10 binds to several sites in the MPZ promoter and enhancers, including a highly conserved element in the first intron of the gene. Dimeric SOX10 binding sites are critical for effective transcriptional activation [103]. EGR2 mediates a substantial upregulation of *MPZ* expression, which is essential for initiating peripheral nerve myelination. The main binding sites of EGR2 are located both in the proximal promoter and in the conserved element of the first intron *MPZ* (intronic enhancer). Still, without SOX10, EGR2 is unable to effectively activate *MPZ* transcription as shown in rat and mouse models.

The S100 calcium-binding protein beta (S100 beta) promoter drives *S100b* gene expression in astrocytes and glial cells in the central and peripheral nervous systems [104]. The S100 beta protein contributes to neurite outgrowth in specific neuronal populations primarily by activating the receptor for advanced glycation end products (RAGE) on neurons. This activation initiates downstream Rac1/Cdc42 and NF-κB signaling pathways, and the subsequent induction of anti-apoptotic genes enhances neuronal survival during development [105,106]. The minimal promoter region, defined as 168 bp upstream of the transcription start site, is sufficient for basal transcriptional activity. Regulatory elements include the enhancer regions located at positions −788/−391 and −1012/−788, which exhibit cell-type-specific activity in the C6 rat glioma cell line. In contrast, the negative regulatory element at −4437/−1012 suppresses promoter activity across various human glioblastoma and neuroblastoma cell lines [107]. When packaged in AAV2/9 or AAV-PHP.eB capsids, the S100 beta promoter drives robust, astrocyte- and glial-specific reporter expression with minimal off-target effects [108].

*SLC1A3* gene encodes the excitatory amino acid transporter 1 (EAAT), a protein highly expressed in the nervous system, with predominant expression in the cerebellum [109]. EAAT1 is crucial for neuroprotection as it mediates the reuptake of synaptic glutamate, thereby preventing its accumulation and subsequent excitoxicity. The promoter of *SLC1A3* gene was characterized by Kim et al. The 2.1-kb regulatory region drives basal activity in various cell types, with the highest activity observed in glial-derived cells, including human glioma H4 cells and primary human astrocytes. This glial-specific enhancement reflects the promoter’s physiological relevance to the central nervous system [110]. Building upon the characterization of the 2.1 kb regulatory region, further analysis mapped the core promoter to a minimal segment between −57 bp and +20 bp relative to the transcription start site (TSS). Unlike typical tissue-specific genes, the EAAT1 promoter lacks tissue-specific enhancers in its immediate flanking region and relies primarily on ubiquitous transcription factors such as Sp1/Sp3 and USF. However, this basal activity can be modulated by physiological and pathological stimuli in a cell-context-dependent manner. This modulation is exemplified in primary human astrocytes, where prolonged treatment with 8-Br-cAMP, EGF, or TGF-α augments EAAT1 promoter activity, mRNA expression, and functional glutamate uptake, whereas TNF-α suppresses these parameters. The EAAT1 promoter nevertheless exhibits significant cell-type-specific regulation. While it functions in various transformed cell lines, its activity is highest in glial cells. Furthermore, upstream regulatory regions mediate negative regulation in primary astrocytes—a feature that is lost in certain glioma cell lines. These observations suggest that post-transcriptional and epigenetic mechanisms are critical for refining EAAT1 expression patterns in vivo.

### 3.3. Precision Gene Therapy via miRNA Control

MicroRNAs (miRNAs) are small, ~23 nucleotide non-coding RNAs that post-transcriptionally regulate gene expression. Through complementary base-pairing with target mRNAs and as a part of the RNA-induced silencing complex (RISC) with Argonaute (Ago) proteins, miRNAs induce mRNA degradation and translational repression [111]. The incorporation of miRNA-binding elements into the expression cassette enables tissue-specific transgene expression, thereby mitigating deleterious off-target effects and organ toxicity. Several investigations suggest that incorporating multiple binding sites for miRNAs expressed in the periphery, but absent from the CNS, can enable the development of CNS-specific gene therapy vectors [112]. MicroRNA-regulated systems for CNS gene therapy might utilize specific miRNAs—such as miR-1, miR-122, miR-124, miR-128, and miR-221—to de-target expression from certain cells and tissues [113]. miRNA-based technologies are essential to enhance the safety and efficacy of AAV gene delivery by mitigating vector-related toxicity [114]. In certain cases, high intravenous doses of AAV have been connected to systemic complications and mortality in humans, mice, and a number of model organisms. Particular attention in clinical trials is paid to liver injury, which is considered to be one of the most frequent and severe side effects [115,116,117].

The decision to incorporate miRNA target sites for restricting *hABCD1* transgene expression must be driven by the risk of off-target toxicity. Furthermore, due to the poorly characterized consequences of *ABCD1* overexpression, studies aiming to suppress it must also evaluate the effects of its ectopic expression in off-target tissues. These assessments should include analysis of cellular stress responses, including apoptosis, necrosis, and other cytotoxicity markers, to verify the intervention’s safety and specificity.

Hordeaux et al. proposed a strategy to reduce gene therapy-mediated toxicity in dorsal root ganglion (DRG) of NHPs, which can cause consequent development of ataxia. Using miR183, they specifically suppressed transgene expression in DRG without affecting transcriptional activity in other cell types. Further studies are needed to evaluate the long-term effects and applicability to other transgenes applied in AAV-based therapies [96]. Several significant complications must be considered in the introduction of microRNA sites into the expression cassette. The inclusion of miRNA-responsive sequences also imposes constraints on the vector’s packaging capacity, reducing the permissible size of the therapeutic transgene. Furthermore, introducing such sequences carries the risk of unintended immunogenicity, thereby potentially activating adverse host immune reactions [94].

Precise spatial control of *hABCD1* transgene expression is a critical determinant of therapeutic efficacy and safety following AAV-mediated delivery. This can be achieved through two potent strategies: using enhanced, cell-specific promoters with engineered enhancer elements, or a combinatorial approach that employs a ubiquitous promoter alongside miRNA target sites. The combinatorial strategy leverages robust, broad-driven expression while conferring tissue specificity through post-transcriptional repression by endogenous miRNA. Both mechanisms represent sophisticated methods for silencing expression in off-target cells, thereby enhancing therapeutic safety while ensuring high-level transgene mRNA production within the nervous system.

## 4. Delivery Routes and Ramifications of AAV Delivery to the Nervous System Used in X-ALD

The organ-specific transduction efficiency of AAV vectors, administered via different delivery routes, has been extensively characterized to advance treatments for inherited central nervous system (CNS) disorders. The development of safe and effective gene therapies for these conditions critically depends on the careful selection of appropriate AAV serotypes, capsid modifications, and administration methods, as the chosen delivery route profoundly influences biodistribution patterns, cellular tropism, and ultimately therapeutic efficacy. This necessitates comprehensive preclinical optimization to achieve optimal target tissue transduction while minimizing potential off-target effects. Several principal routes of administration were being employed in AAV-mediated gene therapy for CNS disorders. The most sufficient among these include direct infusion into cerebrospinal fluid or into the vein, each route presenting distinct advantages in terms of biodistribution, transduction efficiency, and clinical applicability for specific neurological targets [118,119,120].

### 4.1. Direct Infusion of AAV Vectors to the CSF

Cerebrospinal fluid (CSF) is one the most important systems in the brain, providing brain parenchyma with protection, nutrients and removing waste metabolites. It is secreted by the choroid plexus and then circulates within the ventricular system of the brain, subarachnoid cisterns and subarachnoid space, and central canal of the spinal cord [121].

Administration of AAV vectors can be performed directly to the CSF, offering the opportunity of widespread transduction by the vector in CNS, minimising biodistribution across peripheral organs and tissues. Common CSF injection sites include intracerebroventricular (ICV), intracisterna magna (ICM) and lumbar intrathecal (IT) delivery. Each of these sites confers a distinct and characteristic biodistribution profile for the administered vector. This variation arises from the distinct anatomical barriers that the vectors must traverse to reach the brain parenchyma—tight ependymal layer following ICV injection, and pia mater following administration via ICM or IT routes. Direct infusion of vectors into CSF is a highly interventional procedure, and may have various neurosurgical complications when compared to IV delivery. While ICV administration requires precise stereotaxic surgery, ICM injection carries a significant risk of damaging vital brainstem structures and major blood vessels, rendering it clinically unfeasible; consequently, the ICM route is excluded from this review [119,122,123,124,125].

#### 4.1.1. Lumbar Intrathecal (IT) Delivery

Direct delivery of AAV to the CSF can be achieved via lumbar IT injection, typically targeting the intervertebral space typically between L5 and L6. Common delivery methods include acute needle puncture, transient catheterization, or chronic infusion using a catheter connected to an osmotic pump [126,127]. Lumbar intrathecal delivery of AAV bypasses the blood–brain barrier and is a relatively routine, minimally invasive procedure compared to ICM and ICV injections. Lumbar IT administration is highly effective for transducing cells throughout the spinal cord and DRG. This makes it a particularly relevant strategy for treating the myelopathy and peripheral neuropathy associated with AMN [128]. Furthermore, CSF circulation facilitates vector distribution to posterior brain regions, including the cerebellum and brainstem [129]. A key limitation, however, is its variable and often limited transduction of supra-tentorial structures like the cerebral cortex—a primary target in cerebral ALD. Consequently, this uneven distribution poses a significant drawback for treating a global neurodegenerative disease. Intrathecal delivery of AAV vectors has been evaluated in numerous preclinical studies and has demonstrated clinical feasibility in several trials, including a SBT101 gene therapy for patients with AMN (NCT05394064).

#### 4.1.2. Intracerebroventricular (ICV) Delivery

Intracerebroventricular (ICV) injection in mice entails the direct administration of a substance into the cerebral ventricular system, typically facilitated by a stereotaxic apparatus [130]. The procedure involves anesthetizing the animal, making a midline scalp incision, and precisely delivering the injectate into the ventricular space using a specialized needle or micropipette. This method enables broad distribution of the agent throughout the CNS, including the brain, while circumventing the BBB [131,132]. When employing AAV vectors for ICV delivery, widespread transduction can be achieved across CNS tissues. ICV administration provides more consistent and robust transduction of periventricular and supratentorial brain regions than the IT route, offering a potential advantage for treating cerebral ALD, where these areas are primary pathological sites. It effectively targets ependymal cells, neurons, and glia lining the ventricular system, with subsequent diffusion of the vector into the deeper parenchyma. However, its efficacy in transducing the spinal cord is generally lower than that achieved via IT lumbar or ICM routes [133].

Evidence for the limited distribution of ICV administration comes from Meijer et al., who demonstrated in a glioblastoma multiforme model that ICV-delivered AAV vectors led to predominantly localized GFP expression near the injection site in adult nude mice [134]. These findings align with and corroborate previous observations of restricted transduction efficiency following ICV delivery in various adult CNS models [135].

### 4.2. Intravenous (IV) Delivery

Intravenous delivery of AAV vectors to the brain offers a non-invasive route for widespread transduction of CNS and peripheral tissues. Owing to the intricate composition of the vasculature system, AAV can reach deeper brain structures. The efficacy of IV delivery of AAV vectors demonstrates considerable variability across different animal models, primarily due to inherent differences in BBB permeability. Nevertheless, systemic AAV administration, based especially on AAV9-derived vectors, has been shown to achieve widespread distribution encompassing both the CNS and peripheral tissues, including the adrenal glands and liver [136,137,138].

This broad biodistribution profile was clearly demonstrated in a comparative study evaluating two main administration routes for delivering the *hABCD1* coding sequence in *Abcd1^−^/Y* mice using AAV9, a serotype particularly noted for its ability to cross the BBB and effectively target CNS tissues when administered intravenously [90]. The significant CNS tropism of AAV9 makes it a promising naturally occurring vector candidate for potential gene therapy applications in both childhood cerebral ALD and AMN, as these disorders primarily involve pathology in CNS axons and their supporting glial cells. However, despite its ability to cross the BBB and transduce brain cells, AAV9 possesses higher affinity to peripheral tissues, such as the liver, heart and adrenal glands. Consequently, the CNS tissues most affected in cALD and AMN may receive the lowest copies of the therapeutic transgene. Engineering specific AAV serotypes for IV delivery that efficiently cross the BBB with reduced off-target tropism would significantly advance gene therapy for neurological disorders [98,139].

### 4.3. Addressing AAV Therapy Safety Concerns

The development of effective and safe AAV gene therapies require careful monitoring and management of delivery method-associated toxicities, particularly hepatotoxicity and DRG toxicity. Immunosuppressants like prednisolone have been used preclinically and clinically in attempts to mitigate this risk, but showed no benefit in non-human primates [96,140].

Hepatotoxicity is one the major concerns in the context of gene therapy, especially when a vector is administered intravenously [97,141,142,143]. High doses of AAV, used in IV delivery, can lead to serious adverse effects and even death in certain cases [140]. In terms of intrathecal administration, it is important to consider that AAV vectors may enter blood circulation through CSF absorption via the arachnoid villi into the venous sinuses [121]. The transduction of peripheral tissues and organs—especially liver, heart and adrenal glands—can be slightly reduced when vector is administered with an osmotic pump instead of bolus delivery [128]. Comparative safety assessments in NHP indicate that both intravenous and intrathecal administration of AAV vectors can induce acute liver injury, an effect associated with transgene expression [140]. While the exposure to hepatotoxicity is generally considered low to moderate following IT delivery, a proof-of-concept study for SBT101 observed transient elevations in alanine aminotransferase (ALT) and aspartate aminotransferase (AST) in several NHPs approximately five days post-injection, with levels normalizing by the terminal sacrifice [144].

Hepatotoxicity, while mediated by an innate immune response to the viral capsid, represents a well-characterized and major dose-limiting concern for systemic AAV therapies. However, this is not the only significant risk. Thrombotic microangiopathy (TMA) has emerged as another critical, life-threatening adverse event observed at high vector doses [145,146]. TMA is a clinical syndrome characterized by microangiopathic hemolytic anemia, thrombocytopenia, and organ injury, often involving the kidneys [147]. The pathophysiology is thought to involve complement activation triggered by the massive antigenic load of the AAV capsid, leading to widespread endothelial damage and microvascular thrombosis [145]. This risk underscores the critical importance of careful dose selection and proactive monitoring for hematological and renal parameters in addition to liver function in clinical trials.

A further concern associated with both intra-CSF and IV AAV administration is dose-dependent toxicity within the sensory neurons of DRG and peripheral nerves [94,96,148,149]. The principle histopathological findings consisted of axonal degeneration or necrosis, mononuclear infiltrates and hypertrophy or hyperplasia of glial satellite cells. These lesions were observed in 83% of NHPs following CSF delivery, compared to only 32% of animals after IV administration [149]. Moreover, the histological signs of DRG injury correlate with serum level of neurofilament light chain (Nfl) [95,150]. The SBT101 safety assessment in NHPs revealed moderate neuronal degeneration, necrosis and mononuclear cell infiltration in the spinal cord, DRG and peripheral nerves [144]. Furthermore, two cases with serious adverse effects of neurological deficits have been reported in clinical studies following AAV therapy [151,152].

These corroborating data highlight the critical importance of the careful design of AAV vectors, as well as the imperative to develop comprehensive frameworks for the surveillance and mitigation of therapy-related adverse effects. The limited efficacy of intravenous delivery, which remains ineffective without capsid engineering, contrasts with the promise of the intrathecal route for X-ALD. This latter strategy achieves a favorable safety-efficacy balance by circumventing liver tropism and providing adequate neurological coverage. Translating this potential, however, is underscored by NHP data demonstrating that clinical success is contingent upon meticulous dose optimization and concomitant immune modulation.

## 5. Engineered AAV Capsids for Concurrent CNS and PNS Therapy in X-ALD

Effective gene therapy for neurological phenotypes of X-ALD requires AAV vectors capable of widespread transduction in both the central nervous system and peripheral nervous system. In terms of targeting the nervous system, AAV serotype transduction efficiency is directly intertwined with the delivery system. The choice of serotype and route of administration (intrathecal or intravenous) critically determines biodistribution, efficacy, and safety [153]. A fundamental constraint on all therapeutic delivery systems targeting the brain is the presence of the highly selective BBB. Only a few naturally occurring serotypes of AAV can enter the brain parenchyma. Of the various AAV serotypes tested, AAV9 demonstrates superior efficiency in crossing the BBB following IV administration, leading to robust transduction of both neuronal and astrocytic populations in mice and NHP models [136,154,155,156], in addition to exhibiting moderate tropism for PNS tissues such as the DRG [133]. AAV9 is the chosen serotype for AAV-based AMN therapy via intrathecal route (NCT05394064). The efficiency of CNS and PNS targeting was proven at the proof-of-concept stage along with the therapeutic effect on VLCFA level in brain, spinal cord and DRG [128,144]. Another natural candidate serotype for neurodegenerative gene therapy is AAVrh10. While it shares the advantageous ability to cross the BBB efficiently after systemic administration, AAVrh10 exhibits a stronger tropism for glial cells in some models and demonstrates a pronounced efficiency in transducing motor neurons within the spinal cord [133,154,155,157].

One of the major concerns in the use of natural occurring serotypes, however, is their broad tropism, which frequently leads to off-target transduction in non-neuronal tissues such as the liver. This can result in dose-limiting toxicity, a concern applicable to both IV and IT administration routes [115,140,158,159,160,161]. In response to the growing need in development of robust, non-invasive AAV vectors for AAV-based gene therapy applications, four primary design strategies have appeared—direct evolution, rational design, in silico design and AI-assisted protein engineering [23,162,163]. To date, directed evolution has emerged as a powerful strategy for generating a diverse array of capsid variants with enhanced tropism to target neural tissues.

Directed Evolution is a library-based screening approach in which random or rational mutational insertion is introduced to a specific site in the viral protein (VP) amino acid sequence [164,165,166,167,168]. Pursuing this strategy, the Gradinaru’s laboratory at the California Institute of Technology (Caltech) developed Cre Recombination–Based AAV Targeted Evolution (CREATE), a platform that leverages Cre-transgenic animals to evolve novel AAV serotypes with enhanced tropism for specific cell types. This system was subsequently refined into multiplexed CREATE (M-CREATE) to further improve its capabilities [169,170,171].

Although AAV-PHP.B and AAV-PHP.eB represent pioneering capsid variants designed for superior CNS transduction in murine models, their application is constrained by inefficient targeting of the PNS [169]. Despite this relatively broad tropism—encompassing neurons, glial cells, and endothelial cells—these serotypes remained ineffective for targeting the PNS, highlighting a persistent gap. To address this limitation, subsequent efforts yielded novel variants, AAV-MaCPNS1 and AAV-MaCPNS2, which were specifically selected for efficient transduction of PNS structures—including the DRG and sciatic nerve. Furthermore, these variants demonstrated significantly reduced off-target transduction in the liver after intravenous delivery in mice [139]. Notably, these PNS-targeted vectors can efficiently cross the BBB, enabling simultaneous transduction of both the PNS and the CNS following intravenous administration in NHPs.

In parallel, the AAV.CAP-B10 and AAV.CAP-B22 variants were developed using the M-CREATE platform to further enhance CNS specificity [172]. These newly engineered capsids ultimately surpassed both AAV9 and AAV-PHP.eB by mediating robust, neuron-specific transgene expression with a significantly improved safety profile, evidenced by minimal hepatic uptake. Importantly, this favorable transduction and safety profile was conserved in NHPs, as demonstrated in the adult marmoset.

Despite the pronounced success of M-CREATE in rodents, its application remains challenging to efficiently transduce CNS in Old World primates (OWP). This is a significant limitation, as OWPs are evolutionarily closer to humans and represent a more physiologically relevant model for preclinical testing [173,174,175]. To overcome this translational barrier, a newest AAV9 derivative, AAV.CAP-Mac, was engineered through direct evolution within the adult marmoset CNS. This novel capsid exhibits a strong bias for neuronal transduction and the lowest recorded affinity for liver tissue among OWPs [176]. Subsequent characterization across various NHPs species and in human induced pluripotent stem cells (iPSC)-derived neuronal culture has confirmed its superior and translatable tropism for higher mammals.

The development of an optimal AAV-based therapy for X-ALD necessitates a vector capable of global CNS dissemination, coupled with effective PNS transduction. While natural serotypes offer a starting point, engineered capsids—especially those evolved in NHP models—hold greater promise for meeting these criteria. Significant barriers to clinical application persist, including interspecies differences in AAV tropism, the strategic optimization of administration routes (contrasting intravenous for broad CNS distribution with intrathecal for focused PNS delivery), and the successful translation of NHP-validated variants to human patients [175]. Therefore, a critical direction for subsequent investigation involves pioneering capsids that concurrently target both nervous systems and evaluating their efficacy through rigorous comparative studies in humanized models.

## 6. In Vitro Models Applied in X-ALD Gene Therapy

The clinical heterogeneity of X-ALD, with a wide range of phenotypes from asymptomatic to severe forms, remains a significant challenge in terms of modelling the disease. Existing mouse models are deficient in reproducing key aspects of human pathology, in particular the variable penetrance and heterogeneity of clinical manifestations. This fundamental limitation poses a significant challenge in the study of pathogenetic mechanisms and the development of therapeutic strategies that are sufficiently effective for the various forms of patients with X-ALD.

Integrating both in vitro and in vivo modeling approaches provides distinct advantages for developing gene therapies toward clinical application. At present, preclinical studies and validation of therapeutic concepts in X-ALD are predominantly conducted on mouse models [90,128]. While rodent models have provided important insights, certain critical features of human disease pathophysiology can show significant differences. These fundamental limitations raise concerns about the relevance of the findings and underscore the necessity to develop more relevant human model systems for preclinical testing.

### 6.1. Fibroblasts as a Primary Model

The study of fibroblasts derived from X-ALD patients has significantly advanced our understanding of the disease’s molecular underpinnings. This model has provided valuable insights into the accumulation of VLCFA and the characterisation of impaired β-oxidation in peroxisomes. It has also facilitated the investigation of several regulatory cascades and potential compensatory mechanisms [177,178]. X-ALD patient fibroblasts are commonly utilized for initial in vitro assessment of transgene expression; however, their diagnostic utility is limited. While valuable for establishing proof-of-concept for genetic correction, this model fails to recapitulate the biology of the cell types most affected in AMN and cALD—namely, oligodendrocytes, astrocytes, and microglia. Furthermore, it does not account for critical translational factors such as tissue-specific transduction, vector tropism, or clinically effective dosing. In the early stages of gene therapy development, different AAV vectors may be used in vitro and in vivo models. Although this experimental diversity can be justified for primary research, further therapeutic advancement requires the standardization of serotypes and promoters to bridge the preclinical-clinical gap. This alignment is often challenging due to the lack of representative model systems. For instance, in a study by Yi Gong et al. [90] gene therapy was administered to *Abcd1^−^/Y-KO* mice using rAAV9-CBA-*hABCD1*, whereas patient fibroblasts were transduced with rAAV2-CMV-*hABCD1*. Thus, the data, though confirming successful transgene expression, are insufficient for extrapolating therapeutic efficacy to target tissues, underscoring the need for validation in more relevant models. Therefore, the fibroblast model has some serious limitations to consider. These include the absence of tissue-specific context, failure to reflect the disease picture, and difficulty in interpreting the results for further therapy.

Despite the theoretical advantages as physiologically relevant systems, the practical use of primary human cell cultures is significantly constrained. Major limitations include ethical considerations, scarce availability of materials—particularly with rare disease—and poor culture stability. The advent of human induced pluripotent stem cells (hiPSC) technology [179] has made it possible to obtain neural cells, microglia, and brain organoids via direct differentiation. This will improve understanding of the pathogenesis of neurological phenotypes of X-ALD on a cellular and molecular level. Furthermore, such models can be used to study the effects of therapeutic agents on different cell populations, as well as to analyse AAV-tropism and efficacy. This approach opens up new perspectives for preclinical studies.

### 6.2. Advancing X-ALD Research with Human iPSC Platforms

Induced pluripotent stem cells (iPSCs) are a valuable tool for in vitro disease modelling stems from their pluripotency—the ability to differentiate into any cell of the body. The production of iPSCs involves the reprogramming of somatic cells through the utilisation of Yamanaka factors. iPSCs retain the donor’s complete genetic profile, including any pathological gene variants. The pluripotency of iPSCs facilitates the generation of human models of various cell types, including astrocytes, oligodendrocytes, and microglia. The differentiation potential can be extended to engineer three-dimensional models of organoids, which more accurately recapitulate tissue architecture.

Studies frequently report no significant difference in VLCFA levels between iPSCs derived from X-ALD patients and those from healthy donors [180,181]. However, a notable exception was reported in study of early-passage ccALD iPSCs, which exhibited significantly elevated VLCFA levels compared to control iPSCs, AMN-iPSCs, and human embryonic stem cells (hESCs) [42]. In contrast, higher levels of VLCFA have been observed in astrocytes, oligodendrocytes and neurons derived from iPSCs of patients with X-ALD, in comparison to levels observed in healthy controls [181,182,183,184]. Furthermore, a significant increase in the levels of VLCFAs was observed in astrocytes derived from iPSCs of patients with cALD when compared to those derived from patients with AMN [182,183,184]. In addition, oligodendrocytes derived from cALD patient iPSCs demonstrated higher VLCFA levels than those derived from AMN patient iPSCs [183]. The iPSC modelling data are in agreement with clinical observations, demonstrating a correlation between the concentration of C26:0 in intact white matter and the severity of the clinical phenotype in X-ALD patients [185].

Although valuable for neurodegenerative disease research, two-dimensional (2D) iPSC-derived cultures possess significant limitations. These limitations encompass a simplified cellular architecture, an absence of the three-dimensional organization characteristic of native brain tissue, and a lack of complex intercellular interactions. Consequently, 2D models cannot recapitulate intricate neuronal-glial networks and exhibit limited physiological relevance due to aberrant signaling pathways and metabolic processes. To overcome these limitations, more sophisticated model systems are required, such as co-cultures of astrocytes and neurons [72], cerebral and spinal organoids [186,187,188,189], and hybrid systems (e.g., organoids with co-cultured microglia) [190,191]. These advanced models enable a more accurate simulation of key pathological processes in X-ALD, such as impaired myelination and neuroinflammation. The advancement of three-dimensional and co-culture models derived from iPSCs provides a novel avenue for investigating X-ALD pathogenesis and developing AAV-based gene therapies. These systems offer a more physiologically relevant microenvironment by replicating critical intercellular interactions, thereby enabling the optimization of therapeutic parameters such as AAV serotypes and promoter selection, and the evaluation of dose-dependent efficacy and safety.

## 7. In Vivo Models Applied in X-ALD Gene Therapy

The development of reliable in vivo models of ALD is crucial for elucidating disease pathophysiology, identifying biomarkers, and evaluating therapeutics. The endeavor, however, is challenged by disease’s pronounced clinical heterogeneity. Although the biochemical hallmark—VLCFA accumulation due to pathogenic variants of *ABCD1*—is well-defined, the resulting clinical manifestations are highly variable, complicating the creation of representative models.

### 7.1. Late-Onset Neuropathy in an ALDP-Deficient Mouse Model with Exon 1 Deletion

A mouse model of AMN was obtained by classical targeting of the *Abcd1* gene in R1 embryonic stem cells [192].

A genomic clone spanning a region from −10 kb upstream of the first exon to +2.4 kb downstream of the second exon was isolated from an isogenic library. For homologous recombination, two cassettes were inserted into the targeting vector: a neo cassette, which replaced the majority of exon 1 (including the start codon and the sequence encoding the first 284 amino acids of ALDP), and a thymidine kinase (*tk*) gene for negative selection. Following the microinjection of recombinant ESC into C57BL/6J blastocysts, highly chimeric male offspring were bred with females. The successful ablation of the *Abcd1* transcript was subsequently confirmed by RT-PCR analyses of the brain, testes, liver, kidneys, and heart.

Beginning in the early postnatal period, these animals exhibit a characteristic metabolic shift, marked by a reduction in peroxisomal β-oxidation of VLCFA to approximately 40% of control levels in the liver and fibroblasts, concurrent with a 70–240% increase in C26:0 concentrations in the brain, spinal cord, and adrenal glands [193]. By the age of three months, a sharp accumulation of C26:0 and even a tenfold increase in C28:0 are detected in the brain and kidneys; the ratio of C26:0/C22:0 increases 4–5 times in the CNS, and 3–4 times in the kidneys. Nevertheless, macro- and ultrastructural pathology of the white matter of the brain, spinal cord, and peripheral nerves is not detected until 6 months of age; the cortical layer of the adrenal glands shows only moderate swelling of cells with rare lipid inclusions.

Electron-microscopy examinations of sciatic-nerve cross-sections in the exon-1-deleted *Abcd1* mouse show a late-onset peripheral pathology. While the nerve maintains a normal ultrastructure with no neurological involvement up to 6 months, marked structural injury emerges by 15–16 months. This is characterized by abnormally thick and irregular compact myelin sheaths, alongside numerous axons exhibiting degenerative swellings or loss. These pathological findings correlate the slowed conduction velocity characteristic of a demyelinating neuropathy in this AMN-like model.

Clinically, the model remains asymptomatic for a long time: no delay in motor development or decrease in endurance is noted for up to six months. The first functional abnormalities are recorded only after 15 months. Neurophysiological measurements show an elongation of the latency of composite muscle potential and a slowdown in sensory conduction, indicating a predominant lesion of myelin. In behavioral tests, the changes become obvious: in the rotarode, 20-month-old knockout mice are held on a rotating rod for less than 20 s, while wild-type animals are held for more than 60 s; in the 15-min open-field test (OFT), their horizontal activity and the frequency of vertical standing (rearing) decrease by more than half by the same age [194].

Thus, the removal of exon 1 in the knockout mouse model completely shuts down the synthesis of ALDP and forms a system that combines an easily measurable early biochemical defect in VLCFA metabolism with a delayed but steadily progressive neurological phenotype. The model provides a substantial therapeutic window, encompassing the period from early metabolic dysfunction to irreversible demyelination, making a highly suitable platform for preclinical therapeutic testing [192,193].

The *Abcd1* exon 1 deletion model, which is deficient in ALDP, exhibits an initial, protracted phase of purely biochemical disturbance before developing a late-onset, progressive neurodegenerative phenotype. This is characterized by motor incoordination, reduced spontaneous activity, and peripheral nerve demyelination, becoming fully apparent between 15–20 months of age.

### 7.2. Targeted Gene Delivery for AMN Model with Deletion of the First Exon

The initial proof-of-concept AAV9-mediated *hABCD1* gene delivery was established in 2015 using the *Abcd1^−^/Y* knockout mouse model of X-linked adrenomyeloneuropathy (AMN) [90]. Vector administration was performed via IV or ICV injection to 6–8 week-old mice. Both routes successfully transduced CNS tissues, although the resulting distribution and expression profiles were highly dependent on the administration pathway. ICV administration yielded a superior hABCD1 protein expression profile in the brain, achieving levels more than twofold higher than those observed with IV delivery. This route also provided a modest advantage in the spinal cord. However, expression was predominantly localized near the injection site and restricted to astrocytes, microglia, and neurons. In contrast, IV administration resulted in a more homogeneous distribution throughout the CNS, transducing all major neural and vascular cell types. Functionally, IV delivery reduced lysophosphatidylcholine C26:0 by approximately 40% in both the brain and spinal cord and decreased total VLCFAs, while ICV administration produced more modest biochemical corrections. Neither route influenced plasma LPC C26:0 levels. Notably, IV administration was associated with a downward trend in glutathione peroxidase-1 expression and normalization of C26:0/C22:0 and C24:0/C22:0 fatty acid ratios, indicating a partial correction of oxidative stress. Collectively, these findings established systemic IV delivery as the superior modality for achieving broad CNS transduction and metabolic correction in this model.

Subsequent research shifted focus to IT administration of AAV9. This approach, which involves direct delivery into the cerebrospinal fluid, demonstrated efficient transduction of spinal cord and DRG neurons while limited peripheral exposure. This provided a targeted strategy for addressing the core spinal pathology of AMN, and the optimization of infusion parameters established a direct translational framework for human application [128].

In 2024, this IT strategy was further developed in *Abcd1^−^/Y* mice using SBT101, an AAV9-*hABCD1* vector [144]. A single IT infusion via osmotic pump mediated widespread transduction of lumbar, thoracic, cervical spinal cord segments, and the DRG, with *hABCD1* mRNA expression demonstrating a clear dose-dependent increase. At the highest tested dose (3.3 × 10^11^ vg/animal), spinal VLCFA levels fell approximately by 25% compared to untreated knockouts (*p* < 0.05), and mitochondrial DNA content increased around 20%, indicating partial metabolic rescue. Immunohistochemistry revealed robust hABCD1 protein expression in spinal motor neurons and astrocytes, with less staining in oligodendrocytes. Dose–response modelling indicated a minimal effective dose of 2.0 × 10^10^ vg/animal, and interspecies scaling projected a human dose of about 1.0–3.0 × 10^14^ vg. Safety studies in NHP models showed no treatment-related mortality or clinical toxicity. These results provided strong preclinical justification for the ongoing PROPEL Phase 1/2 clinical trial.

### 7.3. Double Knockout Abcd1^−^/Y; Abcd2^−^/^−^ Mouse Model

To quantify the compensatory role of Abcd2 following Abcd1 loss, researchers generated a double knockout (DKO) mouse model (*Abcd1^−^/Y*; *Abcd2^−^/^−^*) by crossing *Abcd1^−^/Y* males with *Abcd2^−^/^−^* females, resulting in offspring devoid of both paralogs [195]. By eight months of age, DKO mice exhibited a severe metabolic phenotype. The C26:0 content was elevated 5- to 6-fold in the spinal cord, sciatic nerve, and adrenal glands to wild type controls. Furthermore, the C26:0/C22:0 ratio was increased by nearly an order of magnitude, a finding also reflected in the plasma (0.047 in DKO versus 0.013 in controls). By twelve months of age, pronounced motor deficits emerged in DKO mice, characterized by a threefold reduction in latency to fall on the rotarod and observable gait freezing (“step in place”) and hypolocomotion in the open field test (OFT). In contrast, these neurological symptoms manifest only at 18–20 months in *Abcd1^−^/Y* single-knockout mice. Transmission-electron-microscopy of the spinal cord in DKO mice model demonstrates that axonal damage is the earliest ultrastructural lesion, with myelin degeneration appearing only secondarily; compared with *Abcd1* single knockouts. These changes arise several months sooner and are accompanied by signs of an inflammatory response, highlighting the functional overlap between the two peroxisomal transporters and the accelerated pathogenic cascade triggered when both Abcd1 and Abcd2 are missing. Histological analysis confirmed an accelerated pathogenic cascade: initial axonal damage is followed by active demyelination and increased reactive astro- and microgliosis. By 20 months, perivascular CD3+ T lymphocyte clusters were observed in the white matter of the spinal cord, indicating a significant inflammatory component. Electrophysiology recording presented a slowdown in both motor and sensory conduction, and biochemical analysis reveals pronounced oxidative stress in the nervous tissue and adrenal glands [195]. Thus, the absence of both transporters dramatically accelerates axonal degeneration and enhances metabolic and immune disorders, demonstrating the functional redundancy of *Abcd2* and turning the DKO model as a stringent benchmark for preclinical therapeutic evaluation.

### 7.4. SBT101 Gene Therapy in the Double Knockout Abcd1^−^/Y; Abcd2^−^/^−^ Mouse Model

A 2024 study [144] further evaluated SBT101 in the more severe *Abcd1^−^/Y*; *Abcd2*^−^/^−^ DKO model, which develops earlier and more aggressive axonal degeneration than the single knockout. A single IT dose was delivered via osmotic pump approximately 11 months prior to the study endpoint.

At 18 months of age, vector genomes and high *hABCD1* mRNA levels were still detected in the spinal cord, confirming long-term persistence. VLCFA content in the lumbar spinal cord was reduced by ~30% compared to untreated DKO mice (*p* < 0.05), and TNF-α mRNA levels, elevated in untreated DKO animals, fell by ~40%, approaching wild-type values. Immunohistochemistry analysis demonstrated strong hABCD1 protein expression in ventral horn motor neurons, DRG neurons, and astrocytes, with punctate labeling in microglia. Confocal microscopy analysis confirmed cytoplasmic localization of hABCD1 in neuronal somata and axons.

Functional assessment at 15 months demonstrated that SBT101 treatment nearly doubled grip strength in the four-limb wire-hang test (mean latency: ~16 s versus ~8 s in untreated controls, *p* < 0.01). Corroborating this functional rescue, histological analysis of lumbar spinal cord revealed reduced microgliosis (Iba1+ area fraction decreased by ~35%) and fewer degenerating axons in toluidine blue–stained semi-thin sections.

Together, these data show that a single IT administration of SBT101 in the DKO model achieves durable transgene expression, improves biochemical and inflammatory markers, and delivers measurable structural and functional neuroprotection in the most stringent preclinical model of AMN.

### 7.5. A Novel Abcd1 Exon 3–9 Deletion Model of X-ALD

The traditional knockout of exon-1 *Abcd1* reproduces the accumulation of VLCFA, but demonstrates only a later neurological decrease [192,193,194].

According to the NCBI gene database, the *BCAP31* gene partially overlaps with the first exon of *ABCD1*, and its regulatory region may extend into the second exon of *ABCD1*. Therefore, to create a novel X-ALD model, a ~8.7 kb genomic segment encompassing exons 3–9 of the *Abcd1* gene was deleted in C57BL/6N zygotes using CRISPR/Cas9-mediated genome editing [196]. Microgliosis was elevated in the CNS of treated Δ3–9 *Abcd1^−^/Y* ALD mice at 12 months of age using Iba-1 immunofluorescence and confocal microscopy. NeuN staining revealed a lower overall neuronal density accompanied by clear signs of neuronal damage. In ALD mice, a reduced oligodendrocyte count was observed, possibly reflecting demyelination.

Biochemical analysis revealed a pronounced accumulation of VLCFAs in the brains of ALD mice, with C22:0, C24:0, and C26:0 levels elevated 1.8-, 3.4-, and 31-fold, respectively, and C26:0/C22:0 ratio 20 times higher than controls. This profile confirms a biochemical deficiency analogous to, though less severe than, human ALD. Furthermore, levels of free cholesterol and reactive oxygen species (ROS) were elevated 1.4-fold, identifying them as potential auxiliary biomarkers.

Behavioral analysis using the OFT revealed anxiety-like behavior in 6-month-old ALD mice. These animals exhibited significant thymotaxis, characterized by prolonged movement along the walls, avoidance of the central arena, and increased time spent in the peripheral zone. Researchers performed a more complex motor task based on a test with a rotating rod, and by the age of 10 months, there was a clear imbalance [196].

Thus, mice with a deletion of exons 3-9 of *Abcd1* gene recapitulate the core biochemical hallmark of the disease—pathological VLCFA accumulation—along with associated early motor deficits.

### 7.6. Gene Therapy Applied in the Model with Δ3–9 Abcd1^−^/Y ALD

In further experiments, the same Δ3–9 *Abcd1^−^/Y* line served as the basis for testing the effectiveness of gene therapy. Researchers constructed the LV.*ABCD1* lentiviral vector expressing full-size human *ABCD1* cDNA, and stereotaxically injected it bilaterally into the area of the outer capsule and thalamus of three-month-old animals (7 × 10^5^ TU/µL; 2 µL per site). Immunofluorescence analysis conducted 15 days post-injection revealed robust, supraphysiological ABCD1 protein surrounding the injection sites. Furthermore, vector-derived genetic material was detected in contralateral brain regions, confirming widespread distribution in the absence of neuronal loss, astrogliosis, or microglial activation.

Functionally, the therapy resulted in a stable rescue of early behavioral disorders. Data from the OFT and rotarod showed that motor performance, which declined in untreated control mutants by 6–7 months of age, gradually recovered to wild-type levels in the LV.*ABCD1*-treated group and was maintained throughout the 12 months observation period. Biochemical analysis corroborated the functional improvements, demonstrating a significant post-treatment reduction in the pathologically elevated levels of saturated VLCFAs (as measured by C24:0/C22:0 and C26:0/C22:0 ratios) and free cholesterol in both plasma and brain tissue, with values approaching those of wild-type controls. Concurrently, the treatment presented a favorable safety profile, as evidenced by stable body weight dynamics, low-level vector biodistribution to the spinal cord, and an absence of systemic oxidative stress. Thus, intracerebral administration of the LV.*ABCD1* lentiviral vectors in the early-onset Δ3–9 *Abcd1^−^/Y* model restores the peroxisomal transporter expression, mitigates pathogenic accumulation of VLCFA, and rescues motor deficits without observable toxicity. The results underscore the therapeutic potential of localized lentiviral gene therapy for the cerebral form of X-ALD [196].

### 7.7. CPZ/EAE Abcd1^−^/Y Mouse Model

Until recently, all available lines of *Abcd1*-deficient mouse models recapitulated only late-onset axonal neuropathy, failing to develop the cerebral form of X-ALD characterized by inflammatory demyelination foci and BBB disruption. Consequently, a prevailing hypothesis is that the cerebral phenotype of X-ALD manifests only upon activation of an additional autoimmune stimulus, which triggers an adaptive immune response against myelin.

To detect cerebral inflammation, two stimuli were combined, each of which mimics the suspected triggers of the disease in humans [197]. Eight-week-old males *Abcd1^−^/Y* (C57BL/6) were first fed for two weeks with 0.2% cuprizon, causing oxidative stress and death of oligodendrocytes. On the fifteenth day, the same group was injected with 100 µg MOG_35–55 in complete adjuvant and 200 ng each of Bordetella pertussis toxin, which triggered the classic adaptive immune response against myelin experimental autoimmune encephalomyelitis (EAE). Five weeks post-diet initiation, high-field magnetic resonance tomography revealed T_2_-weighted hyperintensities in the medial corpus callosum. Post-gadolinium T_1_-weighted scans further identified focal contrast enhancements, representing the initial radiological hallmark of expanding lesion in cerebral ALD. Notably, these lesions were both larger and exhibited more pronounced contrast in *Abcd1^−^/Y* mice compared to wild-type controls subjected to the identical protocol.

Histological analysis confirmed concentric demyelination, and massive infiltration of CD68+ microglia/macrophages, as well as dense deposition of fibrin around blood vessels, a complex of signs characteristic of early cALD. Biochemical analysis revealed pronounced oxidative stress (gp91-phox) and overexpression of IL-18, colocalizing with perivascular macrophages/microglia.

Integrating histopathological and functional data, the researchers showed a significant correlation between the T_2_-hyperintense lesion area, the extent of myelin loss, and the clinical EAE score. Furthermore, for every quantitative parameter assessed—including demyelination, astrogliosis, perivascular infiltrates, and IL-18 levels—*Abcd1*-knockout mice with induced EAE presented more severe pathology than wild-type controls subjected to identical two-hit challenges. Thus, for the first time, the described two-stroke protocol translates the inflammatory cerebral phase of ALD into a reproducible animal model, it combines a reliably forming lesion with rupture of the BBB, concentric demyelination, and rich T/B cell infiltration. The model is the first to recapitulate the precise evolution of cerebral inflammation observed in cALD patients. The establishment of this model enables the systematic screening of anti-inflammatory strategies and the investigation of mechanisms to halt lesion progression and promote remyelination—objectives that were previously unattainable with conventional *Abcd1* KO (Table 1) [197].

This aggressive phenotype provides a rigorous platform for validating ideal AAV9-mediated *ABCD1* gene replacement and other therapeutic strategies. A proposed therapeutic protocol involves systemic vector administration at 5–6 weeks of age, followed by a four-week period for transgene expression and VLCFA reduction prior to subjecting the mice to the combined CPZ and EAE challenge. Primary endpoints include lesion volume and BBB integrity via MRI, alongside semi-quantitative histopathological scores for demyelination, astrogliosis, and immune infiltration, complemented by IL-18 and gp91-phox quantification. These read-outs can be directly compared across four experimental cohorts: untreated knockouts, gene-treated knockouts, wild-type mice subjected to CPZ/EAE, and naïve wild-type controls. Successful therapy should compress every parameter toward the wild-type CPZ/EAE range, indicating that restored peroxisomal β-oxidation and reduced oxidative stress blunt the secondary autoimmune cascade and, by extension, lower the risk of converting to the cerebral inflammatory form of X-ALD.

Based on the results of comparing all available lines, the CRISPR model with deletion of exons 3–9 of the *Abcd1* gene remains the most versatile and methodically verified tool: it provides an early, clearly measurable motor phenotype, reproducible accumulation of VLCFA, and predictably reacts to genetic and pharmacological interventions, making it the “gold standard” for preclinical screening. In the near future, this platform could be further strengthened by creating a Δ3–9 *Abcd1^−^/Y*; *Abcd2^−^/^−^* double-knockout line. Such a strain would couple the convenient early read-outs of the Δ3–9 model with the accelerated axono-myelin degeneration and heightened oxidative/immune stress already documented in classical *Abcd1/Abcd2* double knockouts; in practice, this would tighten study timelines and increase assay sensitivity for therapies aimed at halting axonal loss. At the same time, the two-stroke protocol “Cz/EAE” in *Abcd1^−^/Y* mice is still indispensable when research focuses specifically on the cerebral form of the disease.

Simultaneously, the cuprizone and EAE “two-hit” protocol supplies an indispensable complementary tool, by superimposing an acute oligodendrocyte insult and a myelin-directed autoimmune response on *Abcd1* deficiency. This model successfully recapitulates the inflammatory cerebral phase of X-ALD, a pathology that standard knockout lines fail to develop. This model is therefore ideal for testing whether AAV-based *ABCD1* gene replacement, anti-oxidants or immune-modulating agents can actively lower the risk of a cerebral conversion-an outcome that cannot be judged in the baseline Δ3–9 strain alone.

The Δ3–9 model sets the primary benchmark for systemic therapeutic strategies, while the cuprizone/EAE paradigm provides a focused window on cerebral inflammation and its prevention; the prospective Δ3–9 *Abcd1^−^/Y*; *Abcd2^−^/^−^* double knockout is poised to integrate the advantages of both, offering an even more stringent proving ground for next-generation gene and drug therapies.

## 8. Conclusions

X-ALD presents a complex challenge due to its dual metabolic and neurodegenerative pathology, necessitating a gene therapy approach that effectively targets the central and peripheral nervous systems. AAV-based gene therapy offers a promising strategy by enabling efficient delivery of functional copy of *ABCD1* to affected neural cells, potentially halting or reversing disease progression. However, its success hinges on multiple critical factors: precise regulation of transgene expression to avoid toxicity while ensuring therapeutic efficacy, optimal AAV serotype selection for broad CNS and PNS transduction, a delivery route (IT or systemic) that balances biodistribution and safety, and robust preclinical validation using physiologically relevant in vitro (iPSC-derived glial cells, 3D models) and in vivo (*Abcd1^−^/Y* KO, *Abcd1^−^/Y*; *Abcd2^−^/^−^* DKO, Cz/EAE mice, NHP) models.

While recent advances in AAV engineering and delivery hold great potential, key challenges remain—including off-target effects, immune responses, and the lack of genotype–phenotype correlation, complicating universal therapeutic design. AAV-based approaches must further address the unique demands of neuropathic X-ALD, particularly in achieving sustained VLCFA normalization without liver toxicity. Future research should prioritize combinatorial strategies (e.g., miRNA-mediated regulation, cell-specific promoters) and translational studies in NHPs to bridge the gap between preclinical proof-of-concept and clinical application. Following careful optimization, AAV gene therapy may fundamentally transform the treatment landscape for the neurological sequelae of X-ALD, offering a viable strategy for a disease that currently lacks effective interventions.

## Figures and Tables

**Table 1:** Comparative analysis of developed mice models for X-ALD, their key characteristics and application in gene therapy.

Model (Strain C57BL/6)	Model Generation Method	Key Biochemical Changes	Behavioral Changes/Neurological Manifestation	Tested Gene Therapy and Its Effect
Exon 1 deletion (*Abcd1^−^/Y*) model	Homologous recombination replaced the 5′ part of exon 1 with a neomycin-resistance cassette, completely abolishing ALDP synthesis	Hepatic and peripheral β-oxidation of VLCFA reduced to ~40% of normal; C26:0 rises by 70–240% in brain, spinal cord and adrenals glands during the early postnatal period	Motor coordination and spontaneous activity decline between 15 and 20 months; peripheral nerve-conduction velocity slows from ~15 months	Systemic or intraventricular AAV9-*hABCD1* spreads broadly through the CNS, lowers the C26/C22 ratio by 20–35% and stabilizes motor performance
Double knockout *Abcd1^−^/Y*; *Abcd2^−^/^−^* model	Male *Abcd1* knockouts were crossed with female *Abcd2* knockouts, yielding offspring lacking both peroxisomal transporters	C26:0 in nervous tissue and plasma increases five- to six-fold; the C26/C22 ratio is about ten times higher by eight months	Axonopathy and motor deficit appear as early as 8–12 months of age; time on the rotarod is roughly three times shorter than in wild-type mice	Intrathecal AAV9-*hABCD1* (SBT101) maintains expression for at least 11 months, decreases VLCFA and TNF-α, and doubles fore-limb grip strength
Δ 3–9 deletion (*Abcd1* Δ3–9) model	CRISPR/Cas9 excised exons 3–9 (~8.7 kb) in zygotes; fusion of exon 2 to exon 10 was confirmed and ALDP expression is absent.	C26:0 is 31-fold higher; the C26/C22 ratio is 20-fold higher; free cholesterol and reactive oxygen species are 1.4-fold higher by 12 months.	Thigmotaxis in the open-field test is evident by 6 months; imbalance on the rotarod appears by 10 months.	Local lentiviral LV-*hABCD*1 restores ALDP expression where delivered, normalizes VLCFA and cholesterol, and prevents motor decline for at least 12 months.
Cuprizone and EAE “two-hit” *Abcd1^−^/Y* model	Mice receive 0.2% cuprizone for 14 days, followed on day 15 by MOG35–55 immunization with complete Freund’s adjuvant and pertussis toxin, combining oligodendrocyte stress with a myelin-directed immune response.	Baseline VLCFA excess is present; the challenge triggers a surge in IL-18, an increase in gp91-phox and deposition of fibrin around vessels.	By week 5, MRI detects T_2_-hyperintense, gadolinium-leaking lesions in the corpus callosum; tail and hind-limb paresis occurs on days 16–22.	No gene therapy has currently been tested; the model is used to evaluate anti-inflammatory and BBB-protective strategies.

## Data Availability

The data presented in this study are available in Human Protein Atlas at proteinatlas.org, more specifically: https://www.proteinatlas.org/humanproteome/single+cell/single+nuclei+brain/data#brain_regions (accessed on 22 September 2025) and https://www.proteinatlas.org/ENSG00000101986-ABCD1/single+cell (accessed on 22 September 2025).
